# Evaluation of monthly variations in electrometer calibration coefficients using a charge generator for radiation therapy

**DOI:** 10.1007/s12194-024-00830-w

**Published:** 2024-08-01

**Authors:** Motohiro Kawashima, Maria Varnava, Shuichi Ozawa, Hiromitsu Higuchi, Yoshihiko Hoshino, Mutsumi Tashiro

**Affiliations:** 1 Gunma University Heavy Ion Medical Center, 3-39-22 Showa-Machi, Maebashi, Gunma 371-8511 Japan; 2 Department of Radiation Oncology, Graduate School of Biomedical and Health Sciences, Hiroshima University, 1-2-3, Kasumi, Minami-Ku, Hiroshima, 734-8553 Japan; 3 Hiroshima High-Precision Radiotherapy Cancer Center, 3-2-2 Futabanosato, Higashi-Ku, Hiroshima, 732-0057 Japan; 4 RTQM System Inc., 1-11-5 Hikarimachi, Higashi-Ku, Hiroshima, 732-0052 Japan; 5 Gunma University Hospital, 3-39-15 Showa-Machi, Maebashi, Gunma 371-8511 Japan

**Keywords:** Electrometer calibration coefficient, Quality assurance, Self-inspection, Standard dosimetry

## Abstract

Electrometers are important devices that are part of the standard dosimetry system. Therefore, we evaluated the variation of electrometer calibration coefficients (*k*_elec_) over 1 year in this study. We investigated two types of electrometers: a rate mode and an integrate mode. Each electrometer was connected to a charge generator, a constant charge was applied, and *k*_elec_ was determined by measuring the current. The current measurements were repeated once a month. For electrometers with multiple ranges, measurements were taken at low and medium ranges. Almost all *k*_elec_ measurements agreed within 0.2% of the initial measurements. However, the low range of the electrometer with an integrate mode showed seasonal variation, with a variation greater than 0.2%. This study shows that electrometers may exhibit errors that cannot be detected through annual inspections. The importance of quality assurance using a charge generator at one’s own institution was demonstrated.

## Introduction

It is important to perform quality assurance and control of radiation dose in radiation therapy. One of the most important devices is the standard dosimetry system that consists of an ionization chamber, an electrometer, and cables. An international standard, “Medical electrical equipment—Dosimeters with ionization chambers as used in radiotherapy (IEC 60731)” by the International Electrotechnical Commission (IEC), outlines recommendations and performance criteria for electrometers [1]. The performance requirement specified by IEC 60731 has a relative combined uncertainty of 1.6% for ionization chambers and electrometers. However, this value is very large compared to the standard uncertainty associated with the calibration coefficients [2]. Therefore, other countries, such as Japan, United States of America, Canada, and European countries, are also providing services through separate calibrations [3–6]. The introduction of separate calibrations in Japan is expected to stabilize the standard supply system of absorbed dose-to-water calibrations and further reduce uncertainty in important measurements, such as absorbed dose-to-water in radiotherapy.

Manufacturers offer various forms of electrometers. The main charge collection modes of electrometers used as reference class are an integrate mode and a rate mode [6]. In the integrate mode, the charge input from a chamber is stored in a capacitor, and the amount of charge is calculated from the potential difference between the two ends of the charged capacitor. Naturally, since a capacitor is used, charge leakage exists and is greatly affected by humidity and room temperature. In contrast, the rate mode measures the voltage at both ends of the resistor, which changes dynamically. If the sampling period of the analog-to-digital converter is not fast enough for the maximum frequency component in the pulse current, or if resistors with poor characteristics are used, the input current may be underestimated or overestimated [4]. In addition, since the rate mode uses a lot of digital processing, improper digital noise filtering inside the electrometer can lead to erroneous measurement results [4]. Thus, each electrometer has its own merits and demerits. As far as we know, there are no reports on the accuracy of each electrometer.

The electrometer calibration coefficient (*k*_elec_) provided from the separate calibrations should be managed by each institution. The guidelines for electrometers recommend that electrometers should be calibrated and inspected at the time of initial purchase, after repair, when redundancy checks suggest the need, or at intervals not exceeding 2 years [3, 7]. However, a small drift of about 0.2% over a 3-month period was reportedly observed in the *k*_elec_ values of a low range of the integrate mode electrometer [8]. Therefore, we conducted *k*_elec_ measurements using a charge generator over a long period at our institution to evaluate the monthly variation of the *k*_elec_ values for electrometers with integrate and rate modes.

## Materials and methods

### Measurement setup and environments

In this study, we used the same measurement setup as our previous study [8]. However, the measurement interval and duration differ from the previous study. The measurements in this study were taken monthly to identify long-term trends over an 18-month period, as opposed to the 3-month medium-term in the previous study.

We used the RT521R electrometer (RTQM system/EMF Japan) with a built-in electrometer and charge generator, each with Triax Connectors (2 studs). Since charge is the product of current and time, the charge generator can be used as a current source. The experimental setup consisted of connecting the output of the RT521R to the input of an electrometer using a 3 m triaxial cable with Triax connectors. The direct current applied from the output of the charge generator in RT521R was then measured by the electrometer. In addition, when measuring the *k*_elec_ values for RT521R, the input and output on RT521R were connected. The RT521R electrometer with the rate mode and an electrometer with the integrate mode (RAMTEC Duo, Toyo Medic) were used. Since the integrate mode electrometer has multiple ranges, measurements were taken for the low (L) and medium (M) ranges.

All measurements were conducted under the same conditions. The electrometers were used at least 2 h after the power was turned on, and the zero point was adjusted just before conducting the measurements. The room temperature and relative humidity (RH) ranged from 22.0 to 27.0° C and 30 to 70%, respectively, at the time of measurements. In addition, the RT521R electrometer can record internal temperature, current source temperature, and internal RH. In this study, RT521R had an internal temperature of 34.0 ± 1.0° C, a current source temperature of 39.0 ± 1.0° C, and an internal RH of less than 10%. However, these internal values depend on the room temperature where the electrometer is installed.

### Measurement points and determination of electrometer calibration coefficients (*k*_elec_)

The guidelines for electrometers in Japan recommend that measurements to determine the *k*_elec_ should be taken over a wide range of charges based on clinical practice. In this study, the measurement points were the same as the calibration points used by the Accredited Dosimetry Calibration Laboratories (ADCLs) and are summarized in Table 1.

**Table 1:** Summary of the measurement points for each electrometer

Mode of electrometers	Range	Charge (nC)				
Rate	–	± 500	± 200	± 20	± 5	
Integrate	Low	± 10	± 5	± 1		
	Medium	± 500	± 250	± 100	± 50	± 5

At our institution, the *k*_elec_ values were obtained using the data measured by the electrometer and the amount of charge applied from the charge generator. A graph was created by plotting the measured data on the x-axis and the applied charge data on the y-axis. A linear regression analysis was performed, and the *k*_elec_ was obtained from the slope of the linear function.

### Evaluation of measurements

The accuracy of the measurements in this study has been previously reported and is about 0.13% for *k* = 1 (k, coverage factor) [8]. The accuracy of the charge generator was also reported by Kinoshita et al. to be about 0.15% [9]. In this study, we evaluated the differences between the measured and reference data, and time variations.

First, the *k*_elec_ values obtained from the calibration data of ADCLs for both electrometers before starting the measurements of this study were used as a reference. These data were used as the basis for evaluating the 18-month measurements of each electrometer. We also added the *k*_elec_ obtained from the ADCL at 6 and 12 months after the first measurement for the electrometer that exhibited a large variability in the *k*_elec_ values.

## Results

**Fig. 1:**
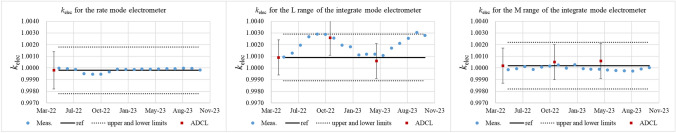
Eighteen-month measurements for each electrometer. The horizontal axis indicates the time of the monthly measurement. The electrometer calibration coefficient (*k*_elec_) values are shown on the vertical axis. Here, the solid line in all figures corresponds to the first *k*_elec_ calculated from the data provided by the Association for Nuclear Technology in Medicine. The upper and lower limits, depicted by dotted lines, incorporate a 0.2% tolerance for long-term stability as outlined in the electrometer guidelines

Eighteen measurements were taken for each electrometer over a 1½-year period. The results are shown in Fig. 1. The *k*_elec_ values obtained from the calibration data provided by the ADCL were added in Fig. 1; for the rate mode, just before the first measurement, and for the integrate mode just before the first measurement, and 6 and 12 months after the first measurement.

The average and standard deviation of the measurements for the first 12 months are summarized in Table 2. In addition, the maximum difference between the *k*_elec_ values obtained from the measurements and the first *k*_elec_ obtained from the ADCL (reference *k*_elec_) was determined for all data and the first 12 months.

**Table 2:** Summary of the data measured over 12 months

		Electrometer calibration coefficients (*k*_elec_)			
Mode of electrometers	Range	Average	Standard deviation	Maximum relative difference* [%]	
				12 months	18 months
Rate	–	0.9998	0.0002	−0.03	−0.03
Integrate	L	1.0019	0.0007	0.20	0.21
	M	1.0000	0.0002	−0.03	−0.05

*(*k*_elec_ from meas. − Reference *k*_elec_)/(Reference *k*_elec_)

**Fig. 2:**
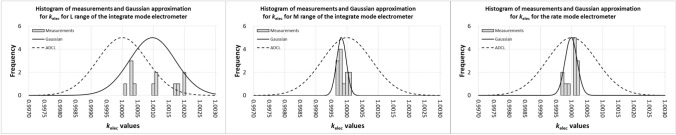
Histograms of measurements and Gaussian approximation of the electrometer calibration coefficient (*k*_elec_) values based on the measurements and data obtained from the ADCL

In addition, histograms of the first 12 measurements for each electrometer are shown in Fig. 2. These data show the relative annual variations, with the reference *k*_elec_ set as 1.0000. An approximation of a Gaussian distribution for each result is also shown, based on the ADCL calibration uncertainty and the average and variation of the measurements.

The Gaussian distributions for the measurements were calculated from the standard deviations and averages in Table 2. In contrast, the Gaussian distributions for the reference *k*_elec_ were calculated with the average set to 1 and the standard deviation obtained from the expanded uncertainty (*k* = 2). The expanded uncertainties were 0.16% and 0.15% for the rate mode and the integrate mode, respectively. The analysis was performed on measurements taken over a 1-year period in order to check for seasonal variations.

## Discussion

The *k*_elec_ values were measured using a charge generator at monthly intervals for 18 months. These data agreed well with the most recent *k*_elec_ values obtained from the ADCL. Therefore, the measurements using the charge generator in the RT521R electrometer could be performed without any issues, and comparison with the ADCL results suggests that they are stable. However, one point obtained from a measurement of the L range of the integrate mode electrometer showed a deviation of 0.21% from the initial ADCL value. The values exceed the long-term stability recommended in the electrometer guidelines. In addition, as can be seen in Fig. 1, seasonal variations were observed for the L range of the integrate mode electrometer within the 18-month period. In contrast, no significant variations were observed for the M range and RT521R, even though the measurements were performed under almost the same conditions. Therefore, these variations are most likely caused by differences in circuitry, especially in the capacitors. It has been reported that there is a correlation between RH and leakage current of capacitors [5]. The integrate mode electrometer uses a capacitor to measure the input charge using a voltage difference. However, the amount of charge handled in the L and M ranges differs by two orders of magnitude. Therefore, it is possible that the L range, which handles minute currents, is greatly affected by temperature and RH, especially because it uses a capacitor with a small capacitance.

The guidelines for electrometers recommend that calibration should be performed at intervals not exceeding a few years [4, 6, 7]. Therefore, the ADCL calibration, which is generally performed at intervals of several years, is not expected to detect these variations. In this study, the *k*_elec_ values obtained from the initial ADCL calibration and the ADCL calibration after 1 year were 1.0009 and 1.0006, respectively, indicating a difference of about 0.03%. This suggests that a once-a-year check may not be sufficient to accurately evaluate electrometer performance.

**Fig. 3:**
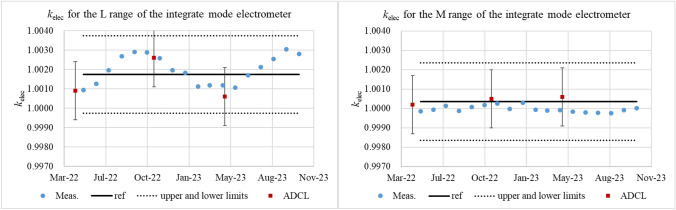
Eighteen-month measurements with shifted reference values

Therefore, we investigated methods that would ensure that all data measured in this study would be stable and within the required 0.2% deviation. We propose a method that averages the *k*_elec_ that is initially measured and the *k*_elec_ that is measured again 6 months later. The *k*_elec_ values of the integrate mode electrometer were obtained three times from the ADCL. The average values when considering the first and second ADCL data are 1.0004 and 1.0018 for the M and L ranges, respectively. The average values when considering the second and third ADCL data are 1.0006 and 1.0016 for the M and L ranges, respectively. The maximum difference between these average values and the data measured in this study was 0.0006 for the M range and 0.0014 for the L range. All data were within 0.2%. This can be seen in Fig. 3. It shows the same measured data as in Fig. 2 for the L and M ranges of the integral mode electrometer, with the baseline changed to the average of the initial ADCL measurements and the ADCL measurements taken after 6 months. This suggests that when determining the initial baseline *k*_elec_, the average of two *k*_elec_ values obtained 6 months apart could be a reliable metric for evaluating the performance of electrometers. However, it may be difficult to calibrate several times a year. In such cases, it is important to manage *k*_elec_ at one’s own institution. A possible alternative, based on the measurements in this study, might be the use of the more stable rate mode electrometer.

We showed that the electrometers with integrate mode may exhibit errors that cannot be detected by the annual inspections recommended by the electrometer guidelines, such as the zero-drift [2, 3]. This is particularly true for the L range, which deals with very small currents. This demonstrates the importance of quality assurance using a charge generator at one’s own institution.

Our study has a few limitations. One limitation is that the electrometers used in this study were one integrate mode and one rate mode. Therefore, the accuracy of all electrometers cannot be determined solely based on this study. We would like to further investigate by increasing the number of units in the future. Moreover, it has already been reported that the accuracy of electrometers is affected not only by the circuitry of the electrometer itself, but also by attachments such as connectors and ionization chamber [2, 5]. The measurements in this study were conducted using the same setup for electrometers so as to eliminate any variations arising from the accessories and other factors.

## Conclusions

The measurements of the *k*_elec_ obtained using a charge generator at our institution were repeated over a period of more than 1 year. Compared to the *k*_elec_ values obtained from the ADCL, stable measurements were obtained without any issues. However, seasonal variations were observed in the *k*_elec_ of the L range of the integrate mode electrometer. Our results suggest that the variation may exceed the annual variation recommended by the electrometer guidelines. This study showed the importance of quality assurance using a charge generator at one’s own institution. Furthermore, it was shown that the electrometers with the rate mode may provide more stable measurements because the electrometers with the integrate mode are affected by environmental factors such as seasonal variations.

## Data Availability

The authors confirm that the data supporting the findings of this study are available within this paper.
